# A Carbon 21 Steroidal Glycoside with Pregnane Skeleton from *Cynanchum atratum* Bunge Promotes Megakaryocytic and Erythroid Differentiation in Erythroleukemia HEL Cells through Regulating Platelet-Derived Growth Factor Receptor Beta and JAK2/STAT3 Pathway

**DOI:** 10.3390/ph17050628

**Published:** 2024-05-14

**Authors:** Jue Yang, Chaolan Pan, Yang Pan, Anlin Hu, Peng Zhao, Meijun Chen, Hui Song, Yanmei Li, Xiaojiang Hao

**Affiliations:** 1 State Key Laboratory of Functions and Applications of Medicinal Plants, Guizhou Medical University, Guiyang 561113, China; yangjue@gmc.edu.cn (J.Y.); panchaolan202312@163.com (C.P.); deer7813py@163.com (Y.P.); anl_hu@163.com (A.H.); zhaopeng960528@163.com (P.Z.); chenmeijunnn@163.com (M.C.); 2 Key Laboratory of Endemic and Ethnic Diseases, Ministry of Education, Key Laboratory of Medical Molecular Biology of Guizhou Province, Guizhou Medical University, Guiyang 561113, China; 3 Natural Products Research Center of Guizhou Province, Guiyang 550014, China; 4 School of Pharmaceutical Sciences, Guizhou Medical University, Guiyang 561113, China; 5 State Key Laboratory of Phytochemistry and Plant Resources in West China, Kunming Institute of Botany, Chinese Academy of Sciences, Kunming 650204, China

**Keywords:** erythroleukemia, natural products, differentiation, PDGFRB, JAK2, STAT3

## Abstract

Erythroleukemia is a rare form of acute myeloid leukemia (AML). Its molecular pathogenesis remains vague, and this disease has no specific therapeutic treatments. Previously, our group isolated a series of Carbon 21 (C-21) steroidal glycosides with pregnane skeleton from the root of *Cynanchum atratum* Bunge. Among them, we found that a compound, named BW18, can induce S-phase cell cycle arrest and apoptosis via the mitogen-activated protein kinase (MAPK) pathway in human chronic myeloid leukemia K562 cells. However, its anti-tumor activity against erythroleukemia remains largely unknown. In this study, we aimed to investigate the anti-erythroleukemia activity of BW18 and the underlying molecular mechanisms. Our results demonstrated that BW18 exhibited a good anti-erythroleukemia activity in the human erythroleukemia cell line HEL and an in vivo xenograft mouse model. In addition, BW18 induced cell cycle arrest at the G2/M phase and promoted megakaryocytic and erythroid differentiation in HEL cells. Furthermore, RNA sequencing (RNA-seq) and rescue assay demonstrated that overexpression of platelet-derived growth factor receptor beta (PDGFRB) reversed BW18-induced megakaryocytic differentiation in HEL cells, but not erythroid differentiation. In addition, the network pharmacology analysis, the molecular docking and cellular thermal shift assay (CETSA) revealed that BW18 could inactivate Janus tyrosine kinase 2 (JAK2)/signal transducer and activator of transcription 3 (STAT3) pathway, which might mediate BW18-induced erythroid differentiation. Taken together, our findings elucidated a novel role of PDGFRB in regulating erythroleukemia differentiation and highlighted BW18 as an attractive lead compound for erythroleukemia treatment.

## 1. Introduction

Erythroleukemia is a subtype of acute myeloid leukemia (AML) and characterized by impaired differentiation and abnormal proliferation of erythroid precursor cells and primordial granulocytes [1,2]. Despite low morbidity, the prognosis of erythroleukemia remains unsatisfactory, with an average survival of only 4–14 months [3]. Current treatments for erythroleukemia include chemotherapy and hematopoietic stem cell transplantation. Given the difficulty in bone marrow matching and high cost, hematopoietic stem cell transplantation is restricted from being widely used in clinical practice. At present, conventional chemotherapy is still the first-line treatment for erythroleukemia. However, due to the lack of high selectivity, traditional chemotherapy drugs can cause serious damage to normal cells and side effects [4]. Therefore, it is necessary to develop effective and non-toxic selective drugs to optimize the erythroleukemia therapy.

Differentiation therapy aims at re-activating the endogenous arrested differentiation programs to induce leukemia cells to differentiate into mature cells [5]. After induction, erythroleukemia cells can undergo erythroid and megakaryocytic differentiation. Hence, differentiation therapy has become one of the promising therapeutic strategies for erythroleukemia [6]. Recently, several studies have reported that natural products can induce differentiation of erythroleukemia. For instance, hirsutine, a tetracyclic heteroyohimbine alkaloid, predominantly found in the *Uncaria* and *Mitragyna* genera, could promote megakaryocytic differentiation by activating the MEK/ERK pathway to up-regulate transcription factors such as *FOG1* and *GATA1* [7]. Song et al. found two novel flavagline-like compounds from plant *Dysoxylum binectariferum* that induced erythroid differentiation through inhibiting oncogene Fli-1 expression in erythroleukemia [8]. All *trans*-retinoic acid (ATRA) is the only approved differentiation inducer for clinical use to treat acute promyelocytic leukemia [9], but it is not applicable to other subtypes. Thus, it is urgent to understand the mechanism involved in erythroleukemia differentiation and identify more effective differentiation inducers.

Carbon 21 (C-21) steroidal glycosides are constituted by pregnane derivatives and 2-deoxy sugars. The hydroxyl group on C-3 is easily connected with the glycosyl group to form the C-21 steroidal glycosides. Pharmacological studies have shown that C-21 steroidal glycosides exhibit a variety of pharmacological activities, including anti-inflammation [10,11], neuroprotection [12], glucose uptake [13] and anti-tumor [14,15]. *Cynanchum atratum* Bunge belongs to the Asclepiadeceae family and is a perennial herb widely distributed in East Asia. The dried root of *Cynanchum atratum* Bunge has several biological effects, including anti-febrility, diuresis and de-intoxication [16]. Studies showed that *Cynanchum atratum* Bunge exerts immunosuppressive, anti-inflammatory and anti-virus activities [17,18,19,20]. Previously, our group isolated a C-21 steroidal glycoside named BW18 from the root of *Cynanchum atratum* Bunge [21]. We found that BW18 induced S-phase cell cycle arrest and apoptosis via the MAPK pathway in human chronic myeloid leukemia K562 cells [22]. However, its anti-tumor activity against erythroleukemia remains largely unknown. 

In this study, we aimed to investigate the anti-erythroleukemia activity of BW18 and related molecular mechanisms. An MTT assay was utilized to evaluate the effect of BW18 on proliferation in erythroleukemia HEL cells. Apoptosis, cell cycle and differentiation of HEL cells treated with BW18 were detected through flow cytometry in vitro. The efficacy of BW18 in vivo was evaluated using a HEL cell xenograft mouse model. RNA sequencing (RNA-seq), network pharmacology analysis and molecular docking were performed to elucidate the possible mechanism. 

## 2. Results

### 2.1. BW18 Exerted Anti-Erythroleukemia Activity in HEL Cells and In Vivo Xenograft Mouse Model

**Figure 1:**
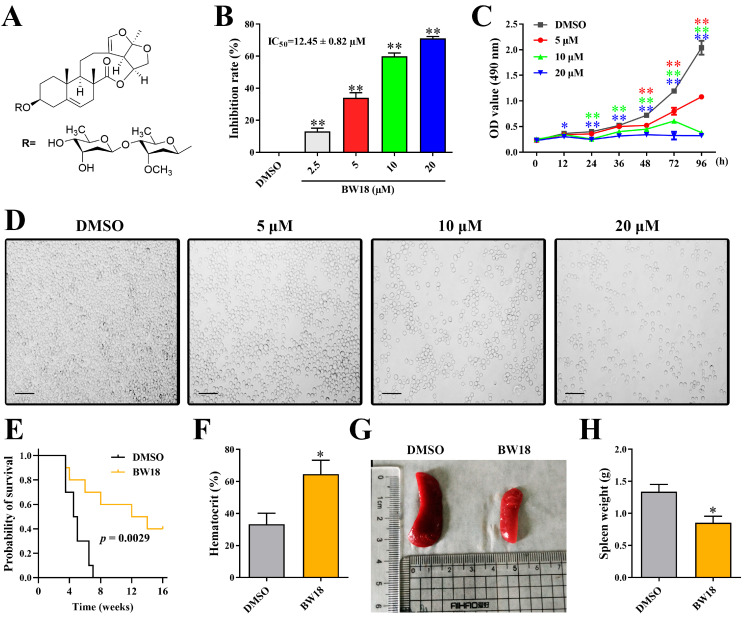
BW18 suppressed the viability and proliferation of HEL cells. (**A**) Chemical structure of BW18. (**B**) HEL cells were treated with various concentrations of BW18 for 72 h. The cell viability was measured using MTT assay. (**C**) The effect of BW18 on HEL cells’ proliferation was determined using MTT assay. (**D**) Effects of BW18 on morphological changes in HEL cells. Magnification: ×200. Scale bar: 100 μm. (**E**) Kaplan–Meier analysis showed that BW18 significantly increased the survival of erythroleukemia mice (*n* = 10). (**F**) The hematocrit values. (**G,H**) Spleen size (**G**) and weight (**H**). Data represent the mean ± SD of three independent experiments. * *p* < 0.05, and ** *p* < 0.01 vs. the DMSO group.

A series of C-21 steroid compounds were previously isolated from the root of *Cynanchum atratum* Bunge by our group (Table 1) [21]. In this study, a 3-(4,5-dimethylthiazol-2-yl)-2,5-diphenyltetrazolium bromide (MTT) assay was first performed to assess the inhibitory effects of these compounds on the viability of erythroleukemia HEL cells. Only BW18 (Figure 1A) exhibited good anti-erythroleukemia activity. After treatment with different concentrations of BW18 (2.5, 5, 10 and 20 µM) for 72 h, the cell viability was dose-dependently reduced, with an IC_50_ value of 12.45 ± 0.82 µM (Figure 1B). Furthermore, the cell growth curve result indicated a pronounced anti-proliferative action of BW18 against HEL cells in a dose- and time-dependent manner (Figure 1C). In addition, microscope observation revealed that BW18 treatment dose-dependently increased cell size and attachment compared to the control group (Figure 1D). In the in vivo xenograft mouse model of erythroleukemia, treatment with BW18 (5 mg/kg) significantly prolonged the survival time of erythroleukemia mice (*p* = 0.0029; Figure 1E). In the meantime, we observed that BW18 dramatically increased hematocrit (Figure 1F) and decreased the spleen size/weight (Figure 1G,H). 

### 2.2. Effects of BW18 on Apoptosis and Cell Cycle Progression in HEL Cells

**Figure 2:**
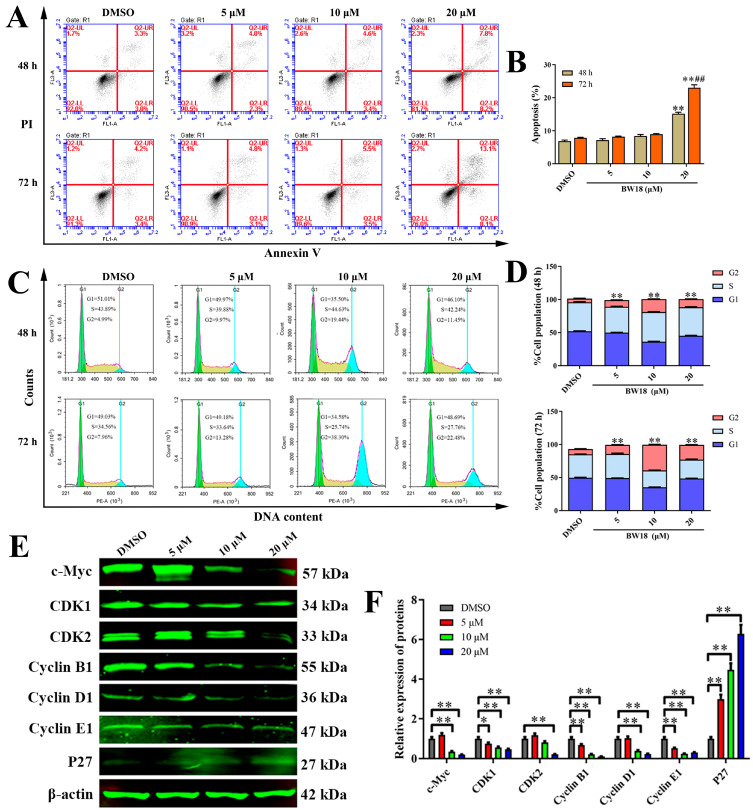
Effects of BW18 on apoptosis and cell cycle progression in HEL cells. (**A**) Annexin V-FITC/PI double staining was used to assess the apoptosis of HEL cells induced by different concentrations of BW18 for 48 h and 72 h. (**B**) Quantification of the apoptosis percentage. (**C**) HEL cells were treated with indicated concentrations of BW18 for 48 h or 72 h. The cells were stained with PI, and the percentage of cell cycle distribution was analyzed by flow cytometry. (**D**) The percentage of cell cycle distribution at G1, S and G2 phases. (**E**) Western blot analysis of cell cycle-related proteins in BW18-treated cells. (**F**) Densitometry analysis of cell cycle-related proteins. All data are expressed as the mean ± SD. β-actin was used as loading control. Each experiment was repeated in triplicate. * *p* < 0.05, and ** *p* < 0.01 vs. the control group. ## *p* < 0.01 vs. BW18-48 h group.

To elucidate the anti-proliferative mechanism of BW18 against HEL cells, the apoptosis and cell cycle progression of BW18-treated HEL cells were analyzed by flow cytometry. After 48 h treatment with different concentrations of BW18 (5, 10 and 20 µM), we observed that only high-concentration BW18 (20 µM) could induce remarkable apoptosis, compared with the control group. The apoptotic cells increased from 6.87% ± 0.55% (DMSO) to 15.17% ± 0.76% (20 µM). The trend of apoptosis rate of BW18-treated HEL cells for 72 h was consistent with that of 48 h, but the apoptosis rate of 72 h was higher than that of 48 h (Figure 2A,B).

The cell cycle analysis demonstrated that treatment with increasing concentrations of BW18 (5, 10, 20 µM) for 48 h significantly enhanced the DNA content of the G2/M phase from 5.33% ±3.04% (DMSO) to 9.94% ± 2.42% (5 µM), 19.29% ± 2.42% (10 µM) and 12.11% ± 2.45% (20 µM) in HEL cells. For 72 h, BW18 treatment also dose-dependently induced cell cycle arrest of HEL cells at G2/M phase (Figure 2C,D). However, the proportion of G2/M phase with high-dose treatment was decreased compared with that if middle-dose treatment, which might be involved in the cell apoptosis induced by high-dose BW18. We also performed Western blot to further assess the effect of BW18 on the expression of cell cycle-related proteins. As shown in Figure 2E,F, BW18 dramatically reduced the expression of c-Myc, CDK1, CDK2, Cyclin B1, Cyclin D1 and Cyclin E1, but increased cyclin-dependent kinase inhibitor P27 level. Taken together, these results imply that BW18 induces G2/M-phase cell cycle arrest and that only a high dose can trigger the apoptosis of HEL cells.

### 2.3. BW18 Induced Megakaryocytic and Erythroid Differentiation in HEL Cells

**Figure 3:**
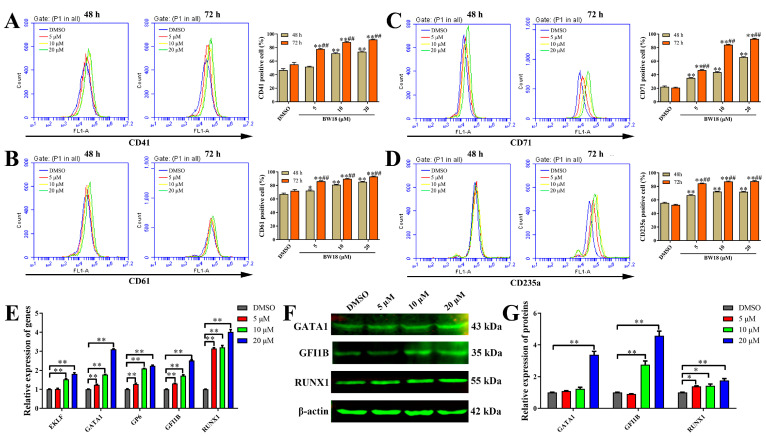
BW18 induced megakaryocytic and erythroid differentiation in HEL cells. (**A**–**D**) Treatment of HEL cells with various concentrations of BW18 increased the percentage of CD41+ cells (**A**), CD61+ cells (**B**), CD71+ cells (**C**) and CD235a+ cells (**D**), both 48 h and 72 h post-treatment. (**E**–**G**) Effects of BW18 on the expression of erythroid differentiation and megakaryocytic differentiation gene in HEL cells. (**E**) mRNA level and (**F**,**G**) protein level. All data are expressed as the mean ± SD. β-actin was used as the loading control. Each experiment was repeated in triplicate. * *p* < 0.05, and ** *p* < 0.01 vs. the control group. ## *p* < 0.01 vs. BW18-48 h group.

To investigate the effect of BW18 on cell differentiation, we determined the expression levels of megakaryocytic (CD41/CD61) and erythroid (CD71/CD235a) differentiation-related markers in BW18-treated HEL cells via flow cytometry. For both 48 h and 72 h, BW18 significantly increased the expression of the megakaryocytic markers CD41/CD61 and erythroid marker CD71/CD235a (Figure 3A–D; Supplementary Figure S1). Longer incubation with BW18 could induce more CD41/CD61- and CD71/CD235a-positive cells. Thus, HEL cells treated with BW18 underwent megakaryocytic and erythroid differentiation in a concentration- and time-dependent manner. In addition, qRT-PCR and Western blot were, respectively, used to determine the effect of BW18 on the mRNA and protein levels of megakaryocytic and erythroid differentiation-related genes in HEL cells. As shown in Figure 3E, BW18 could dose-dependently up-regulate the mRNA levels of megakaryocytic and erythroid differentiation transcription factors *GATA1* and *GFI1B*, as well as erythroid-specific differentiation transcription factor *EKLF*, megakaryocytic-specific differentiation transcription factor *RUNX1* and megakaryocyte marker glycoprotein VI (*GP6*). Consistently, the Western blot results demonstrated that BW18 significantly increased the protein levels of GATA1, GFI1B and RUNX1 (Figure 3F,G). In summary, these results suggest that BW18 induces megakaryocytic and erythroid differentiation in HEL cells.

### 2.4. Platelet-Derived Growth Factor Receptor-Beta (PDGFRB) Was Identified as an Important Downstream Target Gene of BW18

**Figure 4:**
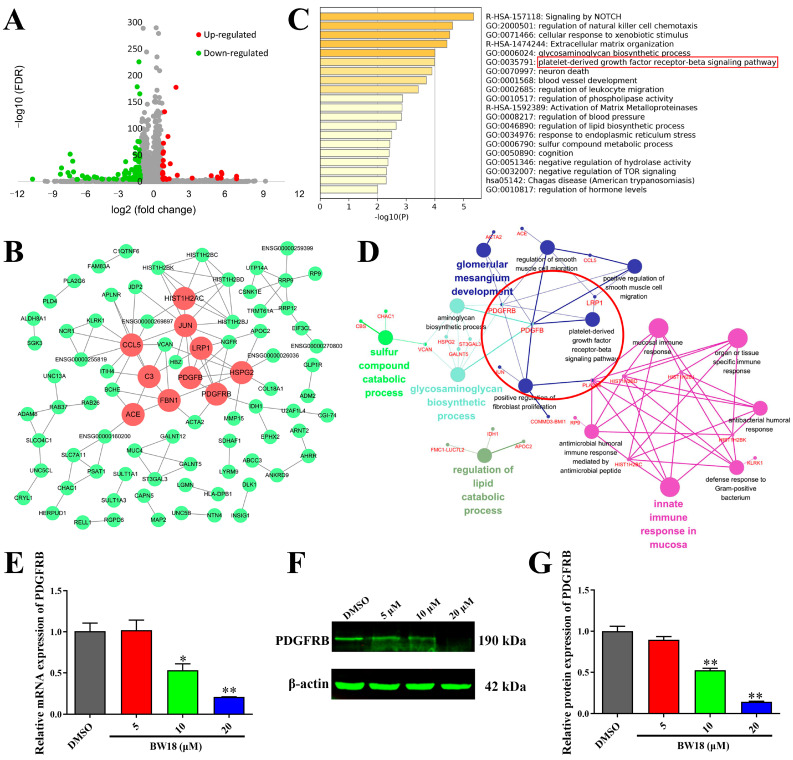
PDGFRB was identified as an important downstream target gene of BW18. (**A**) Volcano plot shows the DEGs between HEL cells treated with and without BW18. Red dots represent up-regulated genes, green dots represented down-regulated genes and grey dots represented unchanged genes. DEGs were identified according to the following criteria: |log2 (fold change)| ≥ 1 and FDR < 0.0001. (**B**) PPI network of 184 BW18-related DEGs. Red represents hub genes. (**C**) Top 20 clusters of enrichment analysis of 184 BW18-related DEGs from Metascape. (**D**) Using ClueGO analysis of 184 BW18-related DEGs, 15 signaling pathways were significantly enriched (*p* < 0.05). (**E**–**G**) Effects of BW18 on expression of PDGFRB in HEL cells. (**E**) mRNA level. (**F,G**) Protein level. All data are expressed as the mean ± SD. β-actin was used as the loading control. Each experiment was repeated in triplicate. * *p* < 0.05, and ** *p* < 0.01 vs. the control group.

To further investigate the underlying mechanisms of the anti-erythroleukemia effect of BW18, we performed RNA-seq in HEL cells treated with BW18 or DMSO for 24 h. As shown in Figure 4A, in total, we obtained 184 BW18-related differentially expressed genes (DEGs), including 29 up-regulated genes and 155 down-regulated genes. We constructed a protein–protein interaction (PPI) network of 184 DEGs (Figure 4B) and then calculated the values of degree, betweenness and closeness of each gene in the network. Through overlapping the top 15 genes with values of degree, betweenness and closeness via a Venn diagram, the genes *JUN*, *CCL5*, *ACE*, *PDGFRB*, *C3*, *HSPG2*, *FBN1*, *LRP1*, *PDGFB* and *HIST1H2AC* were identified as hub genes, which might play an important role in anti-erythroleukemia of BW18 (Supplementary Table S2).

The functional enrichment analysis revealed that 184 BW18-related DEGs were enriched in multiple biological processes and signaling pathways, such as signaling by NOTCH, the glycosaminoglycan biosynthetic process and the PDGFRB signaling pathway (Figure 4C). Consistently, the Gene Ontology (GO) and Kyoto Encyclopedia of Genes and Genomes (KEGG) pathway analysis of these DEGs, using ClueGO, also showed that the glycosaminoglycan biosynthetic process and PDGFRB signaling pathway were strongly associated with the anti-erythroleukemia effect of BW18 (Figure 4D).

To further evaluate the effect of BW18 on the PDGFRB signaling pathway, qRT-PCR and Western blot were performed to detect the mRNA and protein change in PDGFRB. We observed that BW18 treatment could dose-dependently reduce the mRNA level of *PDGFRB* (Figure 4E). Similarly, the Western blot results confirmed that the protein level of PDGFRB was also decreased in HEL cells after treatment with BW18 (Figure 4F,G). Taken together, these results indicate that PDGFRB might be a potential downstream target gene of BW18.

### 2.5. Overexpression of PDGFRB Reversed BW18-Induced Megakaryocytic Differentiation in HEL Cells

**Figure 5:**
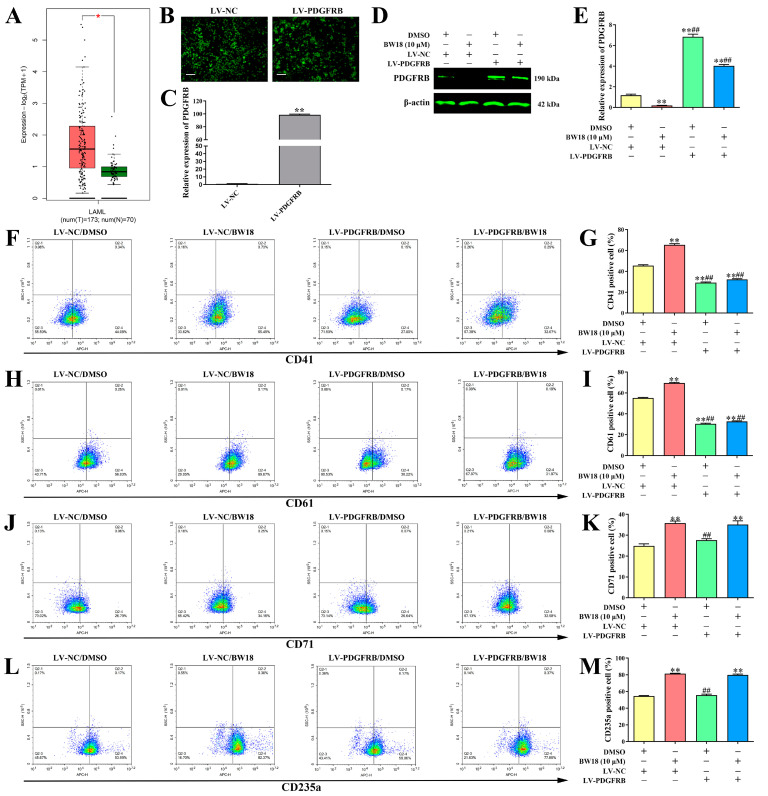
Overexpression of PDGFRB reversed BW18-induced megakaryocytic differentiation in HEL cells. (**A**) The expression level of PDGFRB in AML patients and normal from GEPIA database. (**B,C**) HEL cells were infected with lentiviruses expressing LV-PDGFRB or LV-NC and the overexpression efficiency was then validated by fluorescence microscopy (**B**) and qRT-PCR (**C**). (**D**,**E**) The expression level of PDGFRB, which was suppressed by BW18, was rescued by overexpression of PDGFRB. (**F**–**I**) Moreover, the increase in CD41 (**F**,**G**)/CD61 (**H**,**I**) levels induced by BW18 was completely diminished via PDGFRB overexpression. (**J**–**M**) Overexpression of PDGFRB can’t reverse BW18-mediated up-regulation of CD71 (**J,K**)/CD235a (**L**,**M**) levels. All data are expressed as the mean ± SD. β-actin was used as loading control. Each experiment was repeated in triplicate. **p < 0.01 vs. LV-NC/DMSO group. ## *p* < 0.01 vs. LV-NC/BW18-10 μM group.

The data from GEPIA indicated that the expression level of PDGFRB in AML patients was higher than normal controls (Figure 5A). We then explored whether BW18-induced erythroleukemia differentiation was mediated in a PDGFRB-dependent manner. HEL cells were infected with lentiviruses expressing LV-PDGFRB or LV-NC, and the overexpression efficiency was then validated by fluorescence microscopy and qRT-PCR (Figure 5B,C). Our results demonstrated that the expression level of PDGFRB, which was suppressed by BW18, was rescued by the overexpression of PDGFRB (Figure 5D,E). Moreover, the increase in CD41/CD61 levels induced by BW18 was completely diminished via PDGFRB overexpression (Figure 5F–I). However, overexpression of PDGFRB failed to reverse BW18-mediated up-regulation of CD71/CD235a levels (Figure 5J–M). These results demonstrate that BW18 induces HEL cell megakaryocytic differentiation at least partly by down-regulating PDGFRB expression, but not erythroid differentiation.

### 2.6. BW18 Inactivated Janus Tyrosine Kinase 2 (JAK2)/Signal Transducer and Activator of Transcription 3 (STAT3) Signaling Pathway in HEL Cells

**Figure 6:**
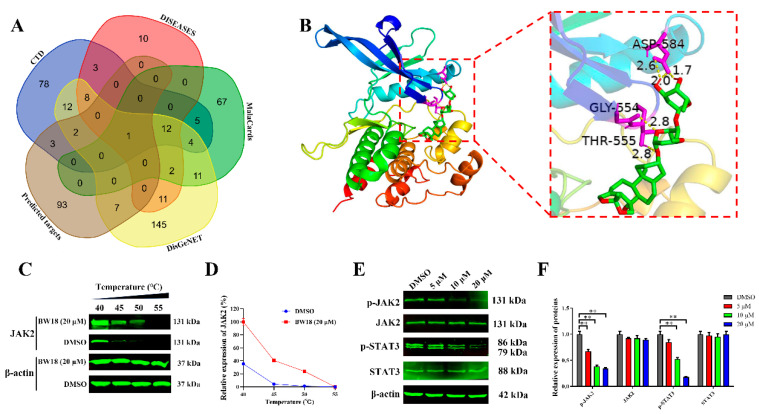
BW18 inactivated JAK2/STAT3 signaling pathway in HEL cells. (**A**) Venn diagram identified JAK2 as the target gene of BW18 in AML by intersecting the predicted targets of BW18 with the targets of AML from different databases. (**B**) AutoDockTools-1.5.6 was used to predict the molecular docking models of BW18 with JAK2. (**C**) CETSA was performed to test the interactions of BW18 and JAK2. (**D**) The graph showed the quantification of JAK2 protein versus temperature points based on Western blot analyses. (**E**) HEL cells were treated with BW18 at the indicated doses for 24 h. The expression levels of p-JAK2, JAK2, p-STAT3 and STAT3 were analyzed by Western blot. (**F**) Densitometry analysis of p-JAK2, JAK2, p-STAT3 and STAT3. All data are expressed as the mean ± SD. β-actin was used as the loading control. Each experiment was repeated in triplicate. ** *p* < 0.01 vs. the control group.

To further investigate the target genes of BW18, the SwissTargetPrediction database was applied to predict the possible targets, and a total of 106 genes were obtained (Supplementary Table S3). The Venn diagram identified JAK2 as the target gene of BW18 in AML through intersecting the predicted targets of BW18 with the targets of AML from different databases (Figure 6A and Supplementary Table S4). Moreover, the results acquired from the molecular docking exhibited that the binding energy of BW18 to JAK2 was -8.81 kcal/mol, indicating a good docking between BW18 and JAK2 (Figure 6B). The Cellular Thermal Shift Assay (CETSA) results showed that BW18 increased the thermal stability of JAK2 compared to the DMSO group at different temperatures (Figure 6C,D). These results suggest that JAK2 is a potential target of BW18. Previous studies from both our group and others have reported that JAK2/STAT3 signaling pathway plays a pivotal role in regulating proliferation and erythroid differentiation of erythroleukemia cells [23,24,25]. Finally, we used Western blot to evaluate the effect of BW18 on JAK2/STAT3 signaling pathway in HEL cells. As displayed in Figure 6E,F, BW18 inactivated JAK2/STAT3 signaling through decreasing the protein expression levels of p-JAK2 and p-STAT3 in a dose-dependent manner. Our data suggest that BW18-induced cell cycle arrest and erythroid differentiation of HEL cells may depend on the inactivation of the JAK2/STAT3 signaling pathway.

## 3. Discussion

In the current study, we explored the anti-erythroleukemia effect of BW18 and found that BW18 significantly inhibited the proliferation of HEL cells by inducing cycle arrest at the G2/M phase and triggering bidirectional differentiation into megakaryocytic and erythroid cells. Furthermore, our data revealed that this efficient pharmacological induction of BW18 was mediated by suppressing the activation of PDGFRB and the JAK2/STAT3 pathway.

In clinical treatment, different subtypes of diseases usually have different responses to the same drug. In breast cancer, for example, a randomized controlled trial suggested that patients with lobular carcinoma are more sensitive to adjuvant letrozole than ductal carcinoma [26]. In leukemia, ETV6-RUNX1-like and ETV6-RUNX1 acute lymphoblastic leukemia showed notable differences in drug sensitivity [27]. Our previous study reported that BW18 can induce S-phase cell cycle arrest and apoptosis via the MAPK pathway in K562 cells [22]. Nevertheless, in this study, we demonstrated that BW18 can induce megakaryocytic and erythroid differentiation, as well as cell G2/M-phase cell cycle arrest in erythroleukemia HEL cells. K562 and HEL cells were derived from patients with chronic myeloid leukemia and erythroleukemia, respectively, which result in diverse leukemia genomics [27,28,29]. This might be responsible for different cellular responses and mechanisms between HEL and K562 cells to the same compound BW18.

Megakaryocyte–erythroid progenitors (MEPs) are a special group of hematopoietic progenitor cells in the bone marrow. MEPs can differentiate into megakaryocyte progenitor and erythroid progenitor cells, which are crucial to the generation of platelets and red blood cells [30,31,32]. Accumulating data have demonstrated that the deregulation of relevant transcription factors, epigenetic modifications and post-translational modifications of proteins can impair the differentiation of MEPs, which are highly associated with the development of erythroleukemia [33,34]. Therefore, it is of great importance for the treatment of erythroleukemia to develop novel strategies to induce the differentiation of erythroleukemia cells into normal cells. Here, our results showed that BW18 significantly increased the expression of the megakaryocytic markers CD41/CD61 and erythroid marker CD71/CD235a, as well as up-regulated expression of differentiation-related transcription factors. Moreover, only the high-concentration BW18 group could induce remarkable apoptosis, indicating a relative low cytotoxicity of BW18 for HEL cells. These results demonstrated that BW18 could promote megakaryocytic and erythroid differentiation in erythroleukemia HEL cells. At present, the majority of differentiation inducers of erythroleukemia cells can only induce either erythroid or megakaryocytic differentiation. It is worth noting that BW18 is a novel bidirectional differentiation inducer of erythroleukemia cells and exhibits great potential as an anti-erythroleukemia lead compound.

An analysis of BW18-related DEGs from RNA-seq suggested that the PDGFRB signaling pathway might be an important mediator for the anti-erythroleukemia of BW18. Simultaneously, our results confirmed that BW18 dramatically decreased the mRNA and protein expression of PDGFRB in HEL cells. The PDGFR family includes two receptor subtypes: PDGFR-α and PDGFR-β. As a typical receptor of tyrosine kinase, PDGFR can induce its tyrosine phosphorylation and regulate signal transduction by binding with corresponding ligands [35]. PDGFR is abnormally activated and acts as a proto-oncogene to regulate the progression of many tumors, including leukemia [36,37,38]. It was reported that PDGFRA inhibited the megakaryocytic and erythroid differentiation through the JNK-JUN-GATA-1 pathway in K562 cells [39]. However, the role of PDGFRB in megakaryocytic and erythroid differentiation has not been reported. In this study, a rescue assay revealed that the overexpression of PDGFRB reversed BW18-induced megakaryocytic differentiation but failed to counteract the erythroid differentiation. Therefore, we highlighted, for the first time, that PDGFRB can regulate megakaryocytic differentiation in erythroleukemia cells. Interestingly, the above-mentioned results provide clear evidence that PDGFRB exerts an inhibitory effect on megakaryocytic differentiation, different from PDGFRA as a bidirectional differentiation inhibitor. Nevertheless, further investigations are needed to clarify the mechanism by which PDGFRB regulates megakaryocytic differentiation.

The JAK2/STAT3 pathway is involved in multiple biological processes, such as cell growth, differentiation, apoptosis and immune regulation [40]. This signaling pathway can transduce signals from the extracellular to the intracellular through the binding of cytokines and growth factors to the corresponding receptors on the cell membrane [41]. The dysregulation of the JAK2/STAT3 pathway is associated with various cancers and autoimmune diseases. Previous studies have shown that the activation of Stat3 could inhibit erythroid differentiation through the up-regulation of Pu.1, thus exacerbating the development of erythroleukemia [24]. Chen et al. found that inhibitors of JAK2 or STAT3 phosphorylation could ameliorate anemia by restoring erythroid cell development [42]. Taken together, these studies suggest that the JAK2/STAT3 pathway plays a key role in regulating erythroid differentiation. Here, both a bioinformatics analysis and experiment validation corroborated that BW18 might inactivate the JAK2/STAT3 pathway through the targeting JAK2. It is well known that JAK2 is a key target for many diseases. Our results suggest that BW18 could serve as an inhibitor of JAK2 and may be utilized to intervene in other diseases caused by JAK2 overactivation.

In conclusion, BW18 exhibited anti-erythroleukemia effects in cell and mouse models.

**Figure 7:**
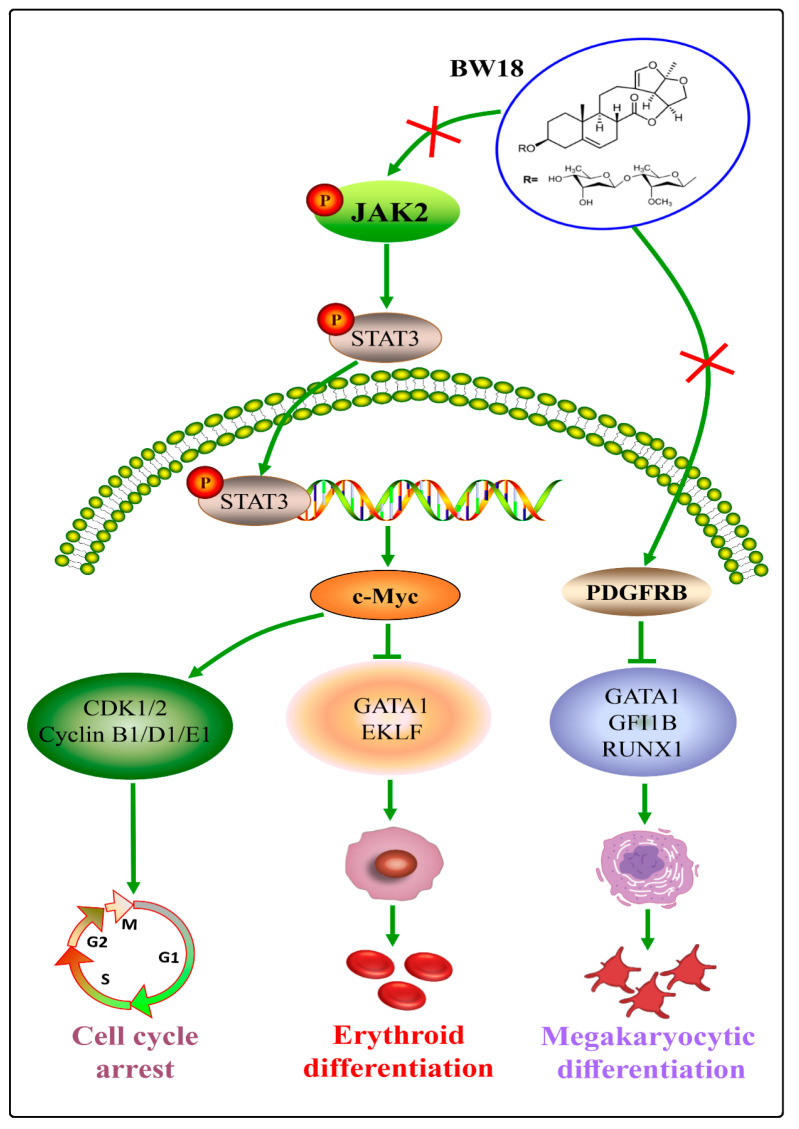
BW18 promotes megakaryocytic and erythroid differentiation in erythroleukemia HEL cells by regulating PDGFRB and JAK2/STAT3 pathway.

In the meantime, our data highlighted BW18 as a novel bidirectional differentiation inducer of erythroleukemia cells to undergo both megakaryocytic and erythroid differentiation. These beneficial effects can be explained by the BW18-mediated inactivation of PDGFRB and JAK2/STAT3 pathway in erythroleukemia cells. Therefore, our research provided a potential lead compound for erythroleukemia differentiation treatment (Figure 7). In the future, efforts to improve the druggability of BW18 are of great significance by chemical structural modification and optimizing drug delivery system. Further investigations are also needed to figure out the mechanism of PDGFRB-mediated megakaryocytic differentiation.

## 4. Materials and Methods

### 4.1. Cell Culture

Human erythroleukemia cell line HEL was obtained from the American Type Culture Collection (ATCC, Manassas, VA, USA) and grown in RPMI 1640 containing 10% fetal bovine serum (FBS, GIBCO, Waltham, MA, USA) at 37 °C in an incubator with 5% CO_2_.

### 4.2. MTT Assay

HEL cells (8 × 10^3^) were seeded in 96-well culture plates and treated with different concentrations of BW18 (white powder, molecular formula: C_34_H_50_O_11_; molecular weight: 634) (2.5, 5, 10, 20 µM) for indicated hours. Then, MTT was applied to BW18-treated and control (0.1% DMSO) group, and the absorbance value at 490 nm was recorded on a multi-well spectrophotometer (BioTek, Winooski, VT, USA).

### 4.3. Apoptosis and Cell Cycle Analysis

HEL cells were seeded in 6-well culture plates at a concentration of 1 × 10^5^ cells/well. Then, cells were harvested after incubating with various doses of BW18 (5, 10 and 20 µM) for 48 h and 72 h. Cell apoptosis and cell cycle analysis were conducted using FACS Calibur flow cytometer (BD Biosciences, Franklin Lakes, NJ, USA) according to a previous description [43].

### 4.4. Assays of Cellular Differentiation

Briefly, for the cellular differentiation assay, the cells were seeded at a concentration of 1 × 10^5^ cells/well in 6-well culture plates and incubated with increasing doses of BW18 (5, 10 and 20 µM) for 48 h and 72 h. Cells were washed and resuspended with 100 µL ice-cold PBS. Anti-CD41-FITC, Anti-CD61-APC, Anti-CD71-FITC and Anti-CD235a-APC (BD Biosciences) were, respectively, added to the cell suspension. After 30 min of incubation on ice, cells were washed and resuspended in 500 μL PBS. Quantification of positive cells was analyzed with FACS Calibur flow cytometer (BD Biosciences).

### 4.5. RNA-Seq and Bioinformatics Analysis

After 24 h treatment with BW18, HEL cells were collected. According to the manufacturer’s instructions, total RNA was extracted by using TRIzol Reagent (Invitrogene, Carlsbad, CA, USA) and RNA-seq was performed on BGI platform (BGI, Shenzhen, China). DEGs were identified based on the criteria |log2 (fold change)| ≥ 1 and false discovery rate (FDR) <0.0001. PPI network of DEGs was constructed using STRING v10.0 [44]. The values of degree, betweenness and closeness of each gene in the PPI network were calculated by the Cytohubba [45]. Venn diagram was used to identify hub genes by overlapping the top 15 genes with values of degree, betweenness and closeness. Functional enrichment analysis of DEGs was conducted using Metascape [46] and the ClueGO [45].

### 4.6. RNA Isolation and qRT-PCR Analysis

After incubating with different doses of BW18 (5, 10 and 20 µM) for 24 h, HEL cells were collected. RNA isolation and qRT-PCR analysis were carried out according to a previous description [43]. Primer sequences are shown in Supplementary Table S1.

### 4.7. Infection of Overexpression Lentivirus and Stable Cell Line Generation

PDGFRB-overexpressed lentiviruses (LV-PDGFRB) were purchased from Genechem company (Shanghai, China). Empty vector lentiviruses expressing GFP (LV-NC) were used as negative control. HEL cells were cultured in 6-well plates and infected with lentivirus particles. The cells were cultured for at least 3 days, and stable cells were selected by puromycin (Solarbio, Beijing, China).

### 4.8. Network Pharmacology Analysis

Disease targets of AML were collected from CTD, DISEASES, MalaCards and DisGeNET. Genes were further selected as disease targets according to the following criteria: genes with direct evidence (marker, mechanism or therapeutic) in CTD, genes with confidence ≥4 stars in DISEASES and genes with score ≥50 in MalaCards. Targets of BW18 were predicted by SwissTargetPrediction (http://www.swisstargetprediction.ch/, accessed on 25 October 2023) [47]. Then, the potential targets of BW18 in AML were obtained using Venn diagram by intersecting disease targets and BW18 targets.

### 4.9. Molecular Docking

The 3D structure of BW18 was generated by KingDraw software v5.0 and saved in mol2 format. The crystal structure of Janus kinase 2 (JAK2, ID: 7F7W) was downloaded from RCSB Protein Data Bank (RCSB PDB) and then dehydrated and hydrogenated using AutoDockTools-1.5.6. The molecular docking between JAK2 protein and BW18 was conducted using AutoDockTools-1.5.6. The molecular docking results were imported and visualized by PyMOL 2.4.1.

### 4.10. Western Blot Analysis

For protein lysates from cells, quantitation and Western blot were performed as described previously [41]. Antibodies used in this study included c-Myc, Cyclin E1, CDK1, CDK2, Cyclin B1, Cyclin D1, PDGFRB, p-STAT3 (Abcam, Cambridge, UK), GATA1, GFI1B, RUNX1, β-actin (CST, Danvers, MA, USA), P27, p-JAK2, JAK2, STAT3 (ZEN BIO, Chengdu, China) and IRDyeR 800CW Goat anti-Rabbit (LI-COR Biosciences, Lincoln, NE, USA).

### 4.11. CETSA

HEL cells were harvested, washed once with PBS and then lysed with PBS buffer with 1 mM protease inhibitor cocktail (Solarbio, Beijing, China). The cells were lysed by three freeze–thaw cycles using liquid nitrogen. Samples were centrifuged at 2000× *g* for 20 min at 4 °C, and the lysates were incubated with BW18 (20 μM) for 1 h at room temperature. Equal amounts of proteins were distributed into PCR tubes and then heated at different temperatures, 40–55 °C. After centrifugation, the soluble supernatants were boiled with a loading buffer for 5 min at 95 °C, followed by Western blot analysis.

### 4.12. In Vivo Xenograft Mouse Model

HEL cells (1 × 10^6^) were injected into the 6-week-old severe combined immunodeficient (SCID) mice (Sibeifu, Beijing, China) through the lateral tail veins. Then, 5 mg/kg BW18 was intraperitoneally injected. Mice showing signs of late-stage disease were sacrificed, and then the percent survival, hematocrit values and spleen weight were calculated. All procedures were approved by the Animal Care and Use Committee of Guizhou Medical University (2304545).

### 4.13. Statistical Analysis

All of the data are presented as the mean ± SD from at least 3 independent experiments. Student’s *t*-test (two-tailed; unpaired) was used to compare the statistical differences between two groups. The one-way analysis of variance (ANOVA) with multiple comparisons test was used to compare the statistical differences between more than two groups. A *p*-value < 0.05 was considered statistically significant.

## Figures and Tables

**Table 1:** IC_50_ values of a series of C-21 steroid compounds in erythroleukemia HEL cells.

Compound	Chemical Structure	IUPAC Name	Chemical Formula	Molecular Weight	IC_50_ (μM)
BW-2	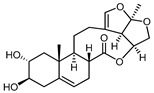	(2aR,2a1R,6aS,6bR,8R,9R,12aR,14aS)-8,9-dihydroxy-2a,6b-dimethyl-1,2a,2a1,5,6,6a,6b,7,8,9,10,12,12a,14a-tetradecahydro-13H-2,3,14-trioxapentaleno[1′,6′:5,6,7]cyclonona[1,2-a]naphthalen-13-one	C_21_H_28_O_6_	376	>20
BW-12	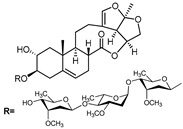	(2aR,2a1R,6aS,6bR,8R,9R,12aR,14aS)-8-hydroxy-9-(((4S,5R,6R)-5-(((2S,4S,5S,6S)-5-(((2S,4S,5R,6R)-5-hydroxy-4-methoxy-6-methyltetrahydro-2H-pyran-2-yl)oxy)-4-methoxy-6-methyltetrahydro-2H-pyran-2-yl)oxy)-4-methoxy-6-methyltetrahydro-2H-pyran-2-yl)oxy)-2a,6b-dimethyl-1,2a,2a1,5,6,6a,6b,7,8,9,10,12,12a,14a-tetradecahydro-13H-2,3,14-trioxapentaleno[1′,6′:5,6,7]cyclonona[1,2-a]naphthalen-13-one	C_42_H_64_O_15_	808	>20
BW-15	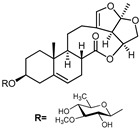	(2aR,2a1R,6aS,6bR,9S,12aR,14aS)-9-(((2R,3R,5R,6R)-3,5-dihydroxy-4-methoxy-6-methyltetrahydro-2H-pyran-2-yl)oxy)-2a,6b-dimethyl-1,2a,2a1,5,6,6a,6b,7,8,9,10,12,12a,14a-tetradecahydro-13H-2,3,14-trioxapentaleno[1′,6′:5,6,7]cyclonona[1,2-a]naphthalen-13-one	C_28_H_40_O_9_	520	>20
BW-17	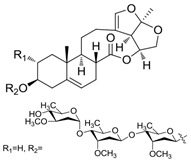	(2aR,2a1R,6aS,6bR,9S,12aR,14aS)-9-(((2R,5R,6R)-5-(((2R,4R,5S,6S)-5-(((2S,4S,5S,6S)-5-hydroxy-4-methoxy-6-methyltetrahydro-2H-pyran-2-yl)oxy)-4-methoxy-6-methyltetrahydro-2H-pyran-2-yl)oxy)-4-methoxy-6-methyltetrahydro-2H-pyran-2-yl)oxy)-2a,6b-dimethyl-1,2a,2a1,5,6,6a,6b,7,8,9,10,12,12a,14a-tetradecahydro-13H-2,3,14-trioxapentaleno[1′,6′:5,6,7]cyclonona[1,2-a]naphthalen-13-one	C_42_H_64_O_14_	792	>20
BW-18	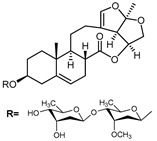	(2aR,2a1R,6aS,6bR,9S,12aR,14aS)-9-(((4S,5R,6R)-5-(((2S,4S,5S,6R)-4,5-dihydroxy-6-methyltetrahydro-2H-pyran-2-yl)oxy)-4-methoxy-6-methyltetrahydro-2H-pyran-2-yl)oxy)-2a,6b-dimethyl-1,2a,2a1,5,6,6a,6b,7,8,9,10,12,12a,14a-tetradecahydro-13H-2,3,14-trioxapentaleno[1′,6′:5,6,7]cyclonona[1,2-a]naphthalen-13-one	C_34_H_50_O_11_	634	12.45 ± 0.82
BW-31	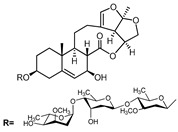	(2aR,2a1R,6aS,6bR,9S,12S,12aS,14aS)-12-hydroxy-9-(((2R,5R,6R)-5-(((2R,4R,5R,6S)-4-hydroxy-5-(((2S,4R,5S,6S)-5-hydroxy-4-methoxy-6-methyltetrahydro-2H-pyran-2-yl)oxy)-6-methyltetrahydro-2H-pyran-2-yl)oxy)-4-methoxy-6-methyltetrahydro-2H-pyran-2-yl)oxy)-2a,6b-dimethyl-1,2a,2a1,5,6,6a,6b,7,8,9,10,12,12a,14a-tetradecahydro-13H-2,3,14-trioxapentaleno[1′,6′:5,6,7]cyclonona[1,2-a]naphthalen-13-one	C_41_H_62_O_15_	794	>20
BW-32	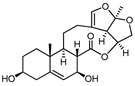	(2aR,2a1R,6aS,6bR,9S,12S,12aS,14aS)-9,12-dihydroxy-2a,6b-dimethyl-1,2a,2a1,5,6,6a,6b,7,8,9,10,12,12a,14a-tetradecahydro-13H-2,3,14-trioxapentaleno[1′,6′:5,6,7]cyclonona[1,2-a]naphthalen-13-one	C_21_H_28_O_6_	376	>20

## Data Availability

Data is contained in the paper.

## Supplementary Materials

The following supporting information can be downloaded at: https://www.mdpi.com/article/10.3390/ph17050628/s1, Table S1. The sequences of primers used in this study. Table S2. The analysis of hub genes. Table S3. Results of predicted targets through SwissTargetPrediction. Table S4. The targets of AML from Database. Figure S1. BW18 induced megakaryocytic and erythroid differentiation in erythroleukemia HEL cells.
