# Supplementary material for: A Carbon 21 Steroidal Glycoside with Pregnane Skeleton from Cynanchum atratum Bunge Promotes Megakaryocytic and Erythroid Differentiation in Erythroleukemia HEL Cells through Regulating Platelet-Derived Growth Factor Receptor Beta and JAK2/STAT3 Pathway
Source: Pharmaceuticals (Basel). 2024 May 14;17(5):628. doi: 10.3390/ph17050628 (PMC11125340; doi:10.3390/ph17050628)
Supplement: Supplementary file 1 [file pharmaceuticals-17-00628-s001.zip › Supplementary Table S1.pdf]

Supplementary Table S1. The sequences of primers used in this study

Genes	Primer sequence
β-actin	Forward primer: 5′-CATGTACGTTGCTATCCAGGC-3′
	Reverse primer: 5′-CTCCTTAATGTCACGCACGAT-3′
GATA1	Forward primer: 5′-TGGTGGCTTTATGGTGGTG-3′
	Reverse primer: 5′-CCTTGGTAGAGATGGGCAGT-3′
EKLF	Forward primer: 5′-GGTTGCGGCAAGAGCTACA-3′
	Reverse primer: 5′-GTCAGAGCGCGAAAAAGCAC-3′
GP6	Forward primer: 5′-CGCTGAACTGACCGTCTCATTC-3′
	Reverse primer: 5′-CAGGTTGCCCTTGGTGTAGTAC-3′
GFI1B	Forward primer: 5′-AGTTCTGCGGCAAGCGTTTCCA-3′
	Reverse primer: 5′-TTTCCGCACACCTGGCACTTGT-3′
RUNX1	Forward primer: 5′-GAGCTTGTCCTTTTCCGAGC-3′
	Reverse primer: 5′-GCACAGAAGGAGAGGCAATG-3′
PDGFRB	Forward primer: 5′-CAAGGACACCATGCGGCTTC-3′
	Reverse primer: 5′-AGCAGGTCAGAACGAAGGTG-3′
