# Supplementary material for: A Carbon 21 Steroidal Glycoside with Pregnane Skeleton from Cynanchum atratum Bunge Promotes Megakaryocytic and Erythroid Differentiation in Erythroleukemia HEL Cells through Regulating Platelet-Derived Growth Factor Receptor Beta and JAK2/STAT3 Pathway
Source: Pharmaceuticals (Basel). 2024 May 14;17(5):628. doi: 10.3390/ph17050628 (PMC11125340; doi:10.3390/ph17050628)
Supplement: Supplementary file 1 [file pharmaceuticals-17-00628-s001.zip › Supplementary Table S2.pdf]

Supplementary Table S2. The analysis of hub genes

Name	Degree	Betweenness	Closeness
JUN	10	457.36667	20.70952
CCL5	9	371.76667	19.9
ACE	4	339.56667	15.9
PDGFRB	6	321.8	18.12619
C3	7	278.26667	18.73333
HSPG2	8	268.46667	18.48333
FBN1	6	198.93333	17.73333
LRP1	5	136.66667	17.45
PDGFB	5	120.86667	17.87619
HIST1H2AC	6	97	16.49286
