# Supplementary material for: A Carbon 21 Steroidal Glycoside with Pregnane Skeleton from Cynanchum atratum Bunge Promotes Megakaryocytic and Erythroid Differentiation in Erythroleukemia HEL Cells through Regulating Platelet-Derived Growth Factor Receptor Beta and JAK2/STAT3 Pathway
Source: Pharmaceuticals (Basel). 2024 May 14;17(5):628. doi: 10.3390/ph17050628 (PMC11125340; doi:10.3390/ph17050628)
Supplement: Supplementary file 1 [file pharmaceuticals-17-00628-s001.zip › Supplementary Table S3.pdf]

Supplementary Table S3. Results of predicted targets through SwissTargetPrediction.

Target	Common name	Uniprot ID	ChEMBL ID	Target Class	Probability	Known actives (3D/2D)
Splicing factor 3B subunit 3	SF3B3	Q15393	CHEMBL1250378	Unclassified protein	0.05564795	9/0
CDK2/Cyclin A	CCNA2<br>CDK2	P20248<br>P24941	CHEMBL3038469	Kinase	0.05564795	41/0
MAP kinase ERK2	MAPK1	P28482	CHEMBL4040	Kinase	0.05564795	185/0
Muscarinic acetylcholine receptor M4	CHRM4	P08173	CHEMBL1821	Family A G protein-coupled receptor	0.05564795	11/0
Muscarinic acetylcholine receptor M5	CHRM5	P08912	CHEMBL2035	Family A G protein-coupled receptor	0.05564795	7/0
Muscarinic acetylcholine receptor M3	CHRM3	P20309	CHEMBL245	Family A G protein-coupled receptor	0.05564795	26/0
Macrophage colony stimulating factor receptor	CSF1R	P07333	CHEMBL1844	Kinase	0.05564795	98/0
Serine/threonine-protein kinase mTOR	MTOR	P42345	CHEMBL2842	Kinase	0.05564795	507/0
Cannabinoid receptor 1 (by homology)	CNR1	P21554	CHEMBL218	Family A G protein-coupled receptor	0.05564795	108/0
Microtubule-associated protein 2	MAP2	P11137	CHEMBL2390810	Unclassified protein	0.05564795	5/0
Protein-tyrosine phosphatase 1B	PTPN1	P18031	CHEMBL335	Phosphatase	0.05564795	248/15
Epidermal growth factor receptor erbB1	EGFR	P00533	CHEMBL203	Kinase	0.05564795	263/0
Dual specificity mitogen-activated protein kinase kinase 1	MAP2K1	Q02750	CHEMBL3587	Kinase	0.05564795	125/0
Tyrosine-protein kinase SYK	SYK	P43405	CHEMBL2599	Kinase	0.05564795	300/0
Sorbitol dehydrogenase	SORD	Q00796	CHEMBL2275	Enzyme	0.05564795	27/0
Interleukin-8 receptor B	CXCR2	P25025	CHEMBL2434	Family A G protein-coupled receptor	0.05564795	37/0
Adenosine A2a receptor	ADORA2A	P29274	CHEMBL251	Family A G protein-coupled receptor	0.05564795	410/0

Cytosolic phospholipase A2	PLA2G4A	P47712	CHEMBL3816	Enzyme	0.05564795	20/0
Cytosolic phospholipase A2 gamma	PLA2G4C	Q9UP65	CHEMBL4834	Enzyme	0.05564795	1/0
Cannabinoid receptor 2	CNR2	P34972	CHEMBL253	Family A G protein-coupled receptor	0.05564795	99/0
DNA-dependent protein kinase	PRKDC	P78527	CHEMBL3142	Kinase	0.05564795	59/0
Sodium/glucose cotransporter 2	SLC5A2	P31639	CHEMBL3884	Electrochemical transporter	0.05564795	379/0
Sodium/glucose cotransporter 1	SLC5A1	P13866	CHEMBL4979	Electrochemical transporter	0.05564795	139/0
MAP kinase p38 alpha	MAPK14	Q16539	CHEMBL260	Kinase	0.05564795	240/0
Cyclin-dependent kinase 4	CDK4	P11802	CHEMBL331	Kinase	0.05564795	83/0
Sphingosine 1-phosphate receptor Edg-3	S1PR3	Q99500	CHEMBL3892	Family A G protein-coupled receptor	0.05564795	80/0
Sphingosine 1-phosphate receptor Edg-1	S1PR1	P21453	CHEMBL4333	Family A G protein-coupled receptor	0.05564795	112/0
Renin	REN	P00797	CHEMBL286	Protease	0.05564795	530/0
Growth factor receptor-bound protein 2	GRB2	P62993	CHEMBL3663	Other cytosolic protein	0.05564795	53/0
Cyclin-dependent kinase 2/cyclin A	CDK2 CCNA1 CCNA2	P24941 P78396 P20248	CHEMBL2094128	Other cytosolic protein	0.05564795	28/0
c-Jun N-terminal kinase 1	MAPK8	P45983	CHEMBL2276	Kinase	0.05564795	100/0
Nerve growth factor receptor Trk-A	NTRK1	P04629	CHEMBL2815	Kinase	0.05564795	135/0
P2X purinoceptor 3	P2RX3	P56373	CHEMBL2998	Ligand-gated ion channel	0.05564795	209/0
Cyclin-dependent kinase 2	CDK2	P24941	CHEMBL301	Kinase	0.05564795	85/0
Cyclin-dependent kinase 1	CDK1	P06493	CHEMBL308	Kinase	0.05564795	77/0
Tyrosine-protein kinase JAK2	JAK2	O60674	CHEMBL2971	Kinase	0.05564795	104/0
Poly [ADP-ribose] polymerase-1	PARP1	P09874	CHEMBL3105	Enzyme	0.05564795	42/0

Gamma-secretase	PSEN2<br>PSENEN<br>NCSTN<br>APH1A<br>PSEN1<br>APH1B	P49810<br>Q9NZ42<br>Q92542<br>Q96BI3<br>P49768<br>Q8WW43	CHEMBL2094135	Protease	0.05564795	45/4
Adenosine A3 receptor	ADORA3	P0DMS8	CHEMBL256	Family A G protein-coupled receptor	0.05564795	333/0
PI3-kinase p110-beta subunit	PIK3CB	P42338	CHEMBL3145	Enzyme	0.05564795	83/0
PI3-kinase p110-alpha subunit	PIK3CA	P42336	CHEMBL4005	Enzyme	0.05564795	400/0
p53-binding protein Mdm-2	MDM2	Q00987	CHEMBL5023	Other nuclear protein	0.05564795	121/0
NAD-dependent deacetylase sirtuin 2	SIRT2	Q8IXJ6	CHEMBL4462	Eraser	0.05564795	4/0
Zinc finger protein GLI1	GLI1	P08151	CHEMBL5461	Transcription factor	0.05564795	0/2
Eukaryotic initiation factor 4A-I	EIF4A1	P60842	CHEMBL2052028	Hydrolase	0.05564795	11/0
Cyclin-dependent kinase 1/cyclin B	CCNB3<br>CDK1<br>CCNB1<br>CCNB2	Q8WWL7<br>P06493<br>P14635<br>O95067	CHEMBL2094127	Other cytosolic protein	0.05564795	12/0
MAP kinase-activated protein kinase 2	MAPKAPK2	P49137	CHEMBL2208	Kinase	0.05564795	21/0
MAP kinase signal-integrating kinase 2	MKNK2	Q9HBH9	CHEMBL4204	Kinase	0.05564795	44/0
Pyruvate dehydrogenase kinase isoform 1	PDK1	Q15118	CHEMBL4766	Kinase	0.05564795	13/0
GABA receptor alpha-5 subunit	GABRA5	P31644	CHEMBL5112	Ligand-gated ion channel	0.05564795	79/0
Calpain 1	CAPN1	P07384	CHEMBL3891	Protease	0.05564795	23/0
Serine/threonine-protein kinase receptor R3	ACVRL1	P37023	CHEMBL5311	Kinase	0.05564795	18/0
Immunoglobulin epsilon Fc receptor	FCER2	P06734	CHEMBL2940	Membrane receptor	0.05564795	5/0

Phosphodiesterase 7A	PDE7A	Q13946	CHEMBL3012	Phosphodiesterase	0.05564795	46/0
Thymidylate synthase (by homology)	TYMS	P04818	CHEMBL1952	Transferase	0.05564795	84/0
Peroxisome proliferator-activated receptor gamma	PPARG	P37231	CHEMBL235	Nuclear receptor	0.05564795	324/0
c-Jun N-terminal kinase 3	MAPK10	P53779	CHEMBL2637	Kinase	0.05564795	33/0
c-Jun N-terminal kinase 2	MAPK9	P45984	CHEMBL4179	Kinase	0.05564795	39/0
Equilibrative nucleoside transporter 1	SLC29A1	Q99808	CHEMBL1997	Electrochemical transporter	0.05564795	85/0
Receptor protein-tyrosine kinase erbB-2	ERBB2	P04626	CHEMBL1824	Kinase	0.05564795	65/0
FK506-binding protein 1A	FKBP1A	P62942	CHEMBL1902	Isomerase	0.05564795	67/0
P2X purinoceptor 7	P2RX7	Q99572	CHEMBL4805	Ligand-gated ion channel	0.05564795	11/0
Arachidonate 5-lipoxygenase	ALOX5	P09917	CHEMBL215	Oxidoreductase	0.05564795	94/0
Protein-tyrosine phosphatase 2C	PTPN11	Q06124	CHEMBL3864	Phosphatase	0.05564795	47/0
Angiotensin II receptor	AGTR2	P50052	CHEMBL4607	Family A G protein-coupled receptor	0.05564795	40/0
CDC7/DBF4 (Cell division cycle 7-related protein kinase/Activator of S phase kinase)	CDC7	O00311	CHEMBL5443	Kinase	0.05564795	8/0
Calmodulin	CALM1	P62158	CHEMBL6093	Unclassified protein	0.05564795	2/0
Corticotropin releasing factor receptor 1	CRHR1	P34998	CHEMBL1800	Family B G protein-coupled receptor	0.05564795	66/0
Mammalian target of Rapamycin (mTORC1)	FKBP1A MTOR	P62942 P42345	CHEMBL2221341	Kinase	0.05564795	13/0
Serine/threonine-protein kinase Chk1	CHEK1	O14757	CHEMBL4630	Kinase	0.05564795	115/0
Complement factor D	CFD	P00746	CHEMBL2176771	Protease	0.05564795	44/0
Leukocyte elastase	ELANE	P08246	CHEMBL248	Protease	0.05564795	29/0
Matrix metalloproteinase 3	MMP3	P08254	CHEMBL283	Protease	0.05564795	117/0

PI3-kinase p110-delta subunit	PIK3CD	O00329	CHEMBL3130	Enzyme	0.05564795	69/0
Matrix metalloproteinase 1	MMP1	P03956	CHEMBL332	Protease	0.05564795	129/0
Adenosine kinase	ADK	P55263	CHEMBL3589	Enzyme	0.05564795	104/0
Matrix metalloproteinase 8	MMP8	P22894	CHEMBL4588	Protease	0.05564795	66/0
Smoothened homolog	SMO	Q99835	CHEMBL5971	Frizzled family G protein-coupled receptor	0.05564795	31/0
Serine/threonine-protein kinase Aurora-B	AURKB	Q96GD4	CHEMBL2185	Kinase	0.05564795	106/0
Inhibitor of nuclear factor kappa B kinase epsilon subunit	IKBKE	Q14164	CHEMBL3529	Kinase	0.05564795	36/0
Serine/threonine-protein kinase TBK1	TBK1	Q9UHD2	CHEMBL5408	Kinase	0.05564795	37/0
Aldehyde dehydrogenase	ALDH2	P05091	CHEMBL1935	Oxidoreductase	0.05564795	4/0
Beta-glucocerebrosidase (by homology)	GBA	P04062	CHEMBL2179	Enzyme	0.05564795	52/0
Phosphodiesterase 4B	PDE4B	Q07343	CHEMBL275	Phosphodiesterase	0.05564795	90/0
Matrix metalloproteinase 2	MMP2	P08253	CHEMBL333	Protease	0.05564795	180/0
Geranylgeranyl transferase type I beta subunit	PGGT1B	P53609	CHEMBL4135	Enzyme	0.05564795	1/0
Oxytocin receptor	OXTR	P30559	CHEMBL2049	Family A G protein-coupled receptor	0.05564795	18/0
Cyclin T1	CCNT1	O60563	CHEMBL2108	Other cytosolic protein	0.05564795	79/0
Mu opioid receptor	OPRM1	P35372	CHEMBL233	Family A G protein-coupled receptor	0.05564795	248/0
Delta opioid receptor (by homology)	OPRD1	P41143	CHEMBL236	Family A G protein-coupled receptor	0.05564795	250/0
Ephrin receptor	EPHB4	P54760	CHEMBL5147	Kinase	0.05564795	31/0
Mitogen-activated protein kinase kinase kinase 14	MAP3K14	Q99558	CHEMBL5888	Kinase	0.05564795	194/0
DNA polymerase alpha subunit	POLA1	P09884	CHEMBL1828	Transferase	0.05564795	1/0

Rho-associated protein kinase 2	ROCK2	O75116	CHEMBL2973	Kinase	0.05564795	71/0
Histone deacetylase 1	HDAC1	Q13547	CHEMBL325	Eraser	0.05564795	120/0
Hepatocyte growth factor receptor	MET	P08581	CHEMBL3717	Kinase	0.05564795	155/0
Acyl-CoA desaturase	SCD	O00767	CHEMBL5555	Enzyme	0.05564795	43/0
Insulin-like growth factor I receptor	IGF1R	P08069	CHEMBL1957	Kinase	0.05564795	102/0
Tyrosine-protein kinase LCK	LCK	P06239	CHEMBL258	Kinase	0.05564795	72/0
Cholesteryl ester transfer protein	CETP	P11597	CHEMBL3572	Other ion channel	0.05564795	1/0
