# Supplementary material for: A Carbon 21 Steroidal Glycoside with Pregnane Skeleton from Cynanchum atratum Bunge Promotes Megakaryocytic and Erythroid Differentiation in Erythroleukemia HEL Cells through Regulating Platelet-Derived Growth Factor Receptor Beta and JAK2/STAT3 Pathway
Source: Pharmaceuticals (Basel). 2024 May 14;17(5):628. doi: 10.3390/ph17050628 (PMC11125340; doi:10.3390/ph17050628)
Supplement: Supplementary file 1 [file pharmaceuticals-17-00628-s001.zip › Supplementary Table S4.pdf]

Supplementary Table S4. The targets of AML from Database.

DISEASES	MalaCards	DisGeNET	CTD
FLT3	CEBPA	CEBPA	FAS
RARA	GATA2	FLT3	BCL2
RUNX1	DNMT3A	NPM1	CASP7
NPM1	JAK2	RUNX1	KIT
CD34	TERT	NUP98	MYC
CD33	FLT3	KMT2A	CCND2
TET2	NPM1	KIT	STAT3
DNMT3A	RUNX1	ABCB1	CDK6
CEBPA	KIT	RUNX1T1	FOXO1
KIT	KIT::MIR221 46	WT1	SGK1
IDH1	KIT::MIR222 46	TBC1D9	BIRC5
ABL1	KRAS	NSD1	TRP53
TP53	ETV6	TET2	SPARC
IDH2	TGM6	VEGFA	RARA
MLLT3	CHIC2	MIR155	CSF2
ASXL1	PICALM	BCR	AGRN
RUNX1T1	MLLT10	BAALC	KMT2A
MYC	NUP214	TP53	CSF1R
CSF3	LPP	WLS	NPM1
BCL2	IDH2	MECOM	AKT1
CD38	TP53	MIR29A	CD44
ANPEP	NRAS	ABCG2	ITGAM
IL3RA	NSD1	IDH2	ANXA2
AKT1	DDX41	MPO	CEBPD
MYH11	SF3B1	ABCC1	DAPK1
WT1	IDH1	CCR7	HSPB1
KMT2A	SRSF2	PTPN11	CTSH
IL3	FANCD2	CD38	CEBPA
HOXA9	BCOR	FANCB	FLT3
JAK2	DNAJC21	MYH11	KIT
NUP98	RTEL1	SF3B1	INPP4B
CD19	INSL6	IL3	TERT
PRAM1	LOC107303338	IDH1	S100A8
NRAS	LOC126807619	SCT	EPHX1
SPI1	LOC126861451	CXXC5	H1-0
SRSF2	MFSD11	KAT6B	MALAT1
CD7	RTEL1-TNFRSF6B	PRAME	RGS2
CBFB	KMT2A	PRDM16	HGF
GATA2	MIR126	VEGFC	ID2
PTPRC	MIR34B	MVP	MX1
CSF2	MIR204	MARCKSL1	RUNX1
ETV6	ERCC6L2	CXCR4	EIF4EBP1

EZH2	MIR320A	ARHGEF1	PML
GATA1	SETBP1	ATM	CAPN2
CD8A	SH3GL1	GLIS2	KRAS
ANXA5	HOTAIR	MIR196B	RUNX3
STAT5B	HOTAIRM1	CSF3	CST3
MCL1	IRAIN	CBFA2T3	ANXA4
U2AF1	UCA1	CREBBP	DNMT3A
MEIS1	CDKN2B-AS1	THPO	IL4R
CD4	CCDC26	NRAS	THBD
NCAM1	CCAT1	DNMT3A	RAC2
STAT5A	TUG1	DNMT3B	ABCA1
DNMT1	WT1-AS	KAT6A	MET
PSMA7	MEG3	CD34	IL17A
ITGAM	MALAT1	MDM2	GLI1
BCL2L1	TUSC7	CD33	IFNG
STAT3	MIR223HG	IKZF1	ENO2
CASP3	MIR142	NCAM1	MN1
KRAS	MIR155	NEO1	PPARG
H3-3B	MIR21	PML	S100A10
H3C12	MIR223	MTTP	ANXA6
KITLG	MIR23A	MIR193B	CD9
GAPDH	MIR125A	PTPRA	GFI1
IL2	MIR27A	SALL4	ATP1B1
H3-5	MIR22	PCBP4	F3
H3C13	MIR335	TRPV2	DHX15
H3-4	MIR23B	SBDS	TCEA2
CTNNB1	MIR221	TBX22	H1-2
H3-2	MIR30A	PAFAH1B1	PDE4B
PML	MIR15B	RTEL1	JAK2
FLT3LG	MIR151A	MIR382	PIM2
MLLT1	MIR34C	MIR375	LEP
BRD4	MIR130B	PLCB4	RASGRP1
BAALC	MIR331	IL17D	MECOM
MPO	MIR324	NTRK1	FHL2
PTEN	MIR146A	ASXL2	DNMT3A
EGFR	MIR222	PAG1	AQP9
MLLT10	MIRLET7B	MAPK3	CAPG
FUT4	MIR210	RARA	MLLT10
IFNG	MIRLET7C	RARRES2	IDH1
MECOM	MIR182	OPN1LW	WT1
CSF3R	MIR128-1	NUP214	TRIO
ZBTB16	MIR424	MLLT10	IFI30
ACTB	MIR195	CALM2	LPAR1
KAT6A	MIR130A	PTP4A2	LPP

CXCR4	MIR328	CALM3	ADCY7
PTPN11	MIR199B	PICALM	NPM1
CD44	MIR181C	CASP1	ANXA5
ERBB2	MIR326	ZNF382	NTRK3
CCND1	MIRLET7E	RUNX3	CTNNA1
PARP1	MIR372	CBFB	RETN
AFF1	MIR30D	ABCC3	ASXL2
CDKN2A	MIR27B	CES2	CTSZ
MDM2	MIR451A	SOCS2	IDH2
IL6	MIR184	ABCC10	VOPP1
ZRSR2	MIR374A	USP10	TRIB3
TNF	MIR340	CD6	ROCK1
CD274	MIR367	CD19	ZBTB7A
RUNX2	MIR302D	ASRGL1	WT1
KLRK1	MIR128-2	CALM1	FLT3
KDM1A	MIR147A	TFPI2	PXDN
CLEC12A	MIR181A2	BCL2	RUNX1T1
HIF1A	MIR325	BCL3	MYH11
CD2		SRSF2	GATA2
SRC		NOM1	PSIP1
FCGR3A		UCA1	NRAS
DEK		SLC25A1	ME1
DOT1L		STAT3	POU4F1
NOTCH1		TERF1	NRAS
BCL2L11		TGFB1	TUBB2A
DNMT3B		FRTS1	EHD3
CD22		TLR2	SEPTIN9
THPO		TLR4	SPRY4
PROM1		MIR663A	TRH
METTL3		U2AF1	ETV6
NUP214		YWHAE	PTPN11
CXCL12		DEK	ZBTB16
FCGR3B		DERL1	EHMT2
MATK		SNAP91	SPI1
IKZF1		GSN	SVIL
HDAC1		SEPTIN6	ERG
ATM		KLRK1	CBFB
CDKN1A		ETV6	DNMT3A
EP300		ABCA3	IDH1
HAVCR2		ERG	BAALC
ABCB1		ERCC2	KMT2C
TET1		ELANE	BACH2
CBFA2T3		EIF4E	RAP1GAP
JUN		EIF4A2	TSC2

BRAF	BRD4	SYNGR1
HLA-A	IL27	ITGB2
CTLA4	ABL1	PTPN11
STAG2	GLI1	DHCR7
IL15	HPGDS	PICALM
LCK	GATA4	GAS2L1
BCR	GATA2	IDH2
CALR	GATA1	DLEU2
CASP9	SETBP1	RAC3
MTOR	CCDC28A	NUMA1
PXDN	EBF3	TET2
PXDNL	FUS	NUP98
EPO	EIF4A1	FXYD6
ELL	ATN1	ENAH
CSF1R	ASXL1	CHMP5
ABCG2	MLLT11	ITGAX
BRCA1	EBP	VSIG4
SETBP1	LINC02682	LYL1
FCGR1A	NCOA2	PRKAR1A
MPL	EFS	SMO
CD47	U2AF1L5	ASMTL
MAPK3	CEBPZ	ITGAL
TAL1	ABCB6	GLI2
RARS1	KLRC4-KLRK1	HOXA9
NFKB1	PIM2	CHIC2
HSP90AA1	PSIP1	DAPK2
CASP8	RHEBL1	LTC4S
GFI1	DDX10	NF1
LOC102723407	ZNF296	LAT2
CYCS	CTSL	CNR2
METTL14	CSF3R	FNDC3B
HSP90AB1	CSF2	GTF2I
CDK2	CSF1R	SH3GL1
XIAP	CCDC26	UMPS
CD5	CPOX	ASXL1
NCR3	CNTFR	KMT2A
CDK4	PDCD6	DHODH
SOX2	GSTM1	CD33
BMI1	ND3	GPHN
PDGFRB	MIR34A	NUP214
COMMD3-BMI1	MIR29C	TNFSF8
ALKBH5	MIR25	MTARC2
ESR1	MIR206	IRF2BP2
JAK1	MIR195	CAD

EGF
DNTT
NANOG
CDK6
IL1B
CD28
TFRC
MYB
PHF6
CEBPE
MME
STUB1
RUNX3
CCK
FTO
GYPA
CHEK1
KLRC1
CDH1
LMO2
CD24
PBX1
ASPG
CREBBP
IFNA1
PDGFRA
POU5F1
IL10
NCOR1
NCR1
E2F1
PAX5
PMAIP1
ALB
CDK9
NTRK1
RARG
NSD1
CDK1
CD48
CDKN2B
RBM15
CD226
hsa-miR-155-5p

MIR192
MIR183
MIR146A
MIR122
MIR34B
MDM4
MEF2C
ATP6
MT3
MSH3
MIR370
MIR342
MIR335
MN1
MME
AFDN
MIR10B
MIR100
LRP1
FAS
IGFBP7
IFNG
HTC2
BIRC5
XIAP
HOXD10
MNX1
HDAC1
IL2
IL2RG
IL3RA
CASC15
RPSA
KRAS
KCNH2
CD82
JAK2
ITGAL
IDO1
IL6
ANXA2

CTCF
DCAF7
DDX41
EZH2
FLT3
HOXB9
KANSL1
KMT2A
NPM1
NRAS
NUP98
RAD21
RB1
RUNX1
STAG2
TP53
TP53
CEBPA
CD44
TNFSF10
CSF3
MIR16-1
NECTIN2
MIR15A
CEBPE
AQP9
SLIT2
DEFA3
PVR
BIRC5

FOXO3
TET3
TERT
STAT1
AFDN
TNFRSF9
PLK1
HOXA10
ITGA2B
PRF1
NF1
KLRD1
TCF3
DDX41
SLAMF1
XPO1
ALK
GSK3B
ITGB3
EPOR
CD86
COLEC10
WTAP
MN1
KIR2DL1
CTCF
NCR2
IL4
YTHDF2
CD80
KDM6A
FASLG
CD247
MYCN
CEBPB
EIF4E
SPN
TNFRSF8
FN1
FOXP3
GZMB
IL7
PDCD1
BRCA2

DCK
BTK
AXL
BRD2
TOP2B
HDAC2
SIRT1
CXCL8
FGFR1
CDKN1B
HOXA7
KDR
SMC3
SUZ12
BRD3
RAD21
FER
JAK3
KLF4
RPS6KB1
BCL6
SNAI1
RARB
PRAME
CHEK2
SIRPA
CCNL2
ABCC1
TGFB1
PIK3CA
YTHDF1
MMP9
ALDH1A1
hsa-miR-34a-5p
SETD2
THY1
H4C6
ASB2
GFI1B
MAP2K1
hsa-miR-21-5p
ITGAX
H4C7
LYN

CEP70
KDM5A
H2BC21
FBXW7
LAG3
HRAS
SYK
DIABLO-2
KLRC2
KIR3DL1
IL7R
CREB1
TOP2A
DIABLO
SMC1A
TCHP
MLF1
CCR5
HLA-C
AURKB
DICER1
CCNA1
CD96
TNFRSF10B
FOXO1
FANCA
IDO1
FOS
CCNB1
CD79A
hsa-miR-29b-3p
KMT2C
TIGIT
HDAC6
EHMT2
FIP1L1
CCNA2
AGO2
IGF1R
NCOA2
CDA
GLIS2
CRBN
RAD51

AURKA
YTHDC1
BRDT
H2AC18
KLF1
GLI1
hsa-miR-223-3p
FGF2
EPCAM
H2AC20
BCL2L2
EWSR1
BCORL1
IRF8
CD40
FOXM1
AR
ZEB1
SDC1
EVPL
CSF1
LYL1
EGR1
HOXA5
NCOR2
CCND2
MICB
CD276
IL1A
PRMT5
SMO
EIF4EBP1
ETS1
RPS14
LGR5
HSPA4
hsa-miR-15a-5p
TNFSF10
SMARCA4
SH2B3
BECN1
LEF1
CD36
ICAM1

SHH
GATA3
ALKBH1
CD27
MDM4
FAS
CDH2
PDCD1LG2
NCL
hsa-miR-125b-5p
SCARB2
VIRMA
B2M
RELA
CYP3A4
SCARB1
hsa-miR-181a-5p
FANCD2
H2AX
hsa-miR-146a-5p
MSI2
LAMP1
NFKBIA
SOCS1
FANCC
ANXA8
HNRNPC
ASXL2
ULBP1
hsa-miR-29a-3p
CBL
PBX3
KIR2DL3
TCL1A
LGALS9
CD68
WEE1
TNFSF9
MIR17HG
CD52
SDHC
KAT2B
hsa-miR-126-3p
SELL

JAG1
MET
GRB2
IFNA2
HLA-E
VAV1
ATRX
NPTN
HLA-B
CD69
MX1
YTHDC2
hsa-miR-17-5p
HOXD13
HNF4A
PIM2
CBFA2T2
CCL2
NECTIN2
PKM
RHOA
FH
PVR
CENPV
CFLAR
hsa-miR-150-5p
BCL2A1
HOXB4
MUC1
HDAC4
ELANE
TRIB2
RNF4
APAF1
PIK3CD
SUV39H1
F3
MMP2
TLR4
CD1D
SNAI2
IL18
ZAP70
NSD2

RCOR1
EIF2AK3
hsa-miR-20a-5p
DNMT3L
CDKN3
EPAS1
CD9
KAT5
MMUT
KDM4C
FANCG
IL17A
SBDS
RET
INS
TNFRSF4
PPARG
IGF1
BRD9
PPM1D
CD1C
hsa-miR-145-5p
TCL1B
IRF1
CCND3
TP53BP1
HOXA4
CDC42
B3GAT1
IFNA17
RAF1
SLCO6A1
CDK7
hsa-miR-221-3p
SOCS2
FLT1
BIRC2
ATG7
YTHDF3
KDM4A
MLLT11
DAXX
KAT6B
MGMT

CD40LG
NEDD8
YY1
IFNA13
MAPK1
ANKRD26
DROSHA
HNMT
Hsa-let-7a-5p
CTAG1B
TDG
NES
LDHA
TRIM24
TNFRSF10A
DHODH
IFNA14
MAPK8
hsa-miR-193a-3p
ERG
STK11
IFNA8
IFNA10
KDM6B
CCR7
IFNA6
IFNA7
SLC2A1
TRIB1
IFNA5
IFNA4
PRMT1
IFNA16
IFNA21
CRYGC
ANXA2
SMAD2
CSNK1A1
CCL5
SUMO1
KAT2A
MAPRE2
ATG5
IRF4

NUMA1
SMAD3
HDAC8
CD70
PHGDH
INVS
WDR5
REL
PECAM1
DAPK1
AANAT
AARS1
ABCA3
