# Supplementary figures and images for: A Carbon 21 Steroidal Glycoside with Pregnane Skeleton from Cynanchum atratum Bunge Promotes Megakaryocytic and Erythroid Differentiation in Erythroleukemia HEL Cells through Regulating Platelet-Derived Growth Factor Receptor Beta and JAK2/STAT3 Pathway
Source: Pharmaceuticals (Basel). 2024 May 14;17(5):628. doi: 10.3390/ph17050628 (PMC11125340; doi:10.3390/ph17050628)

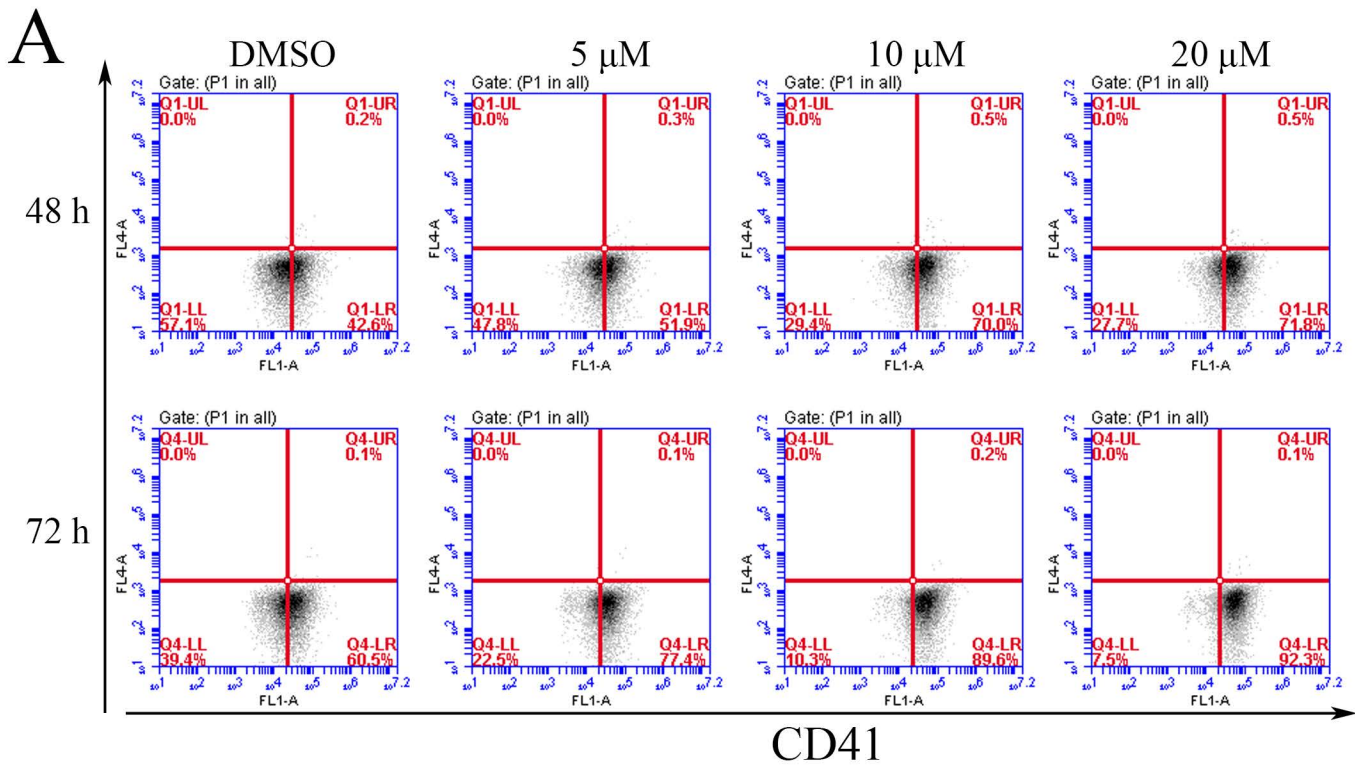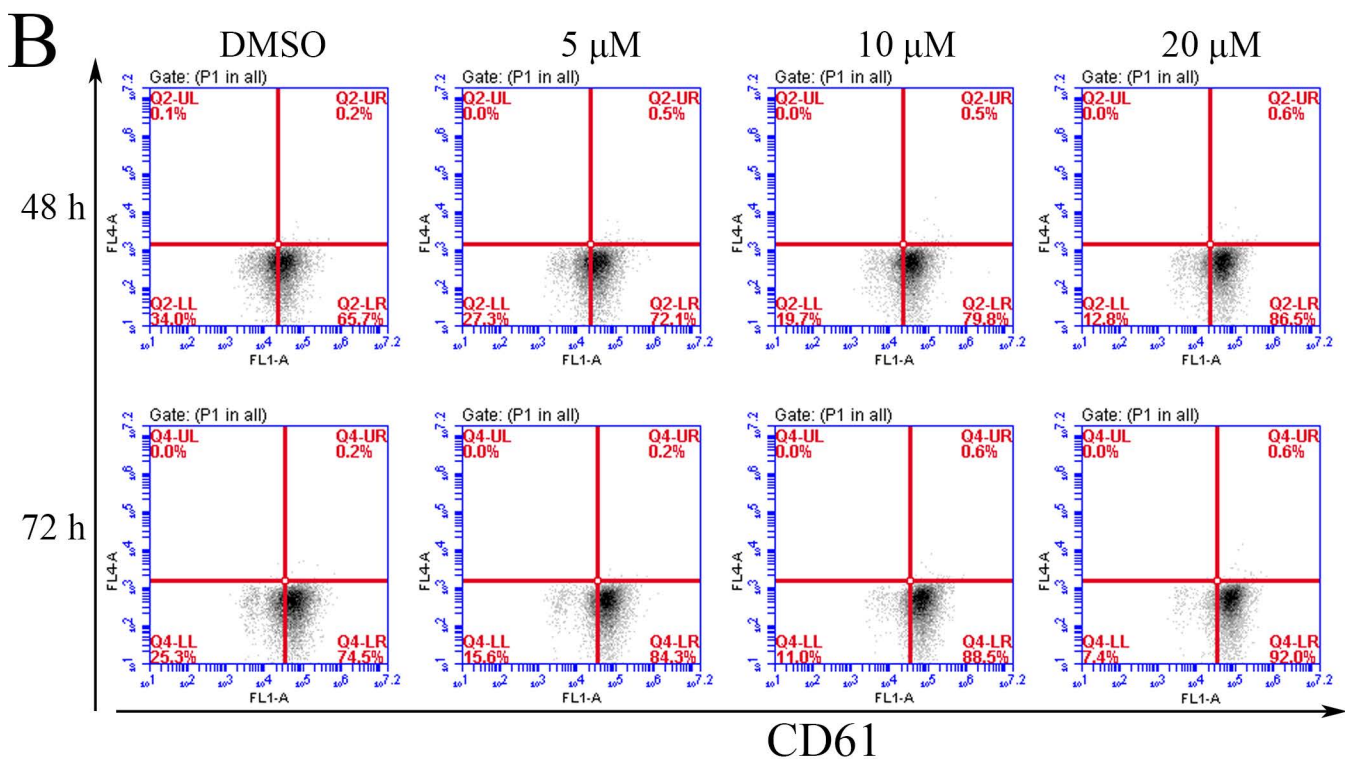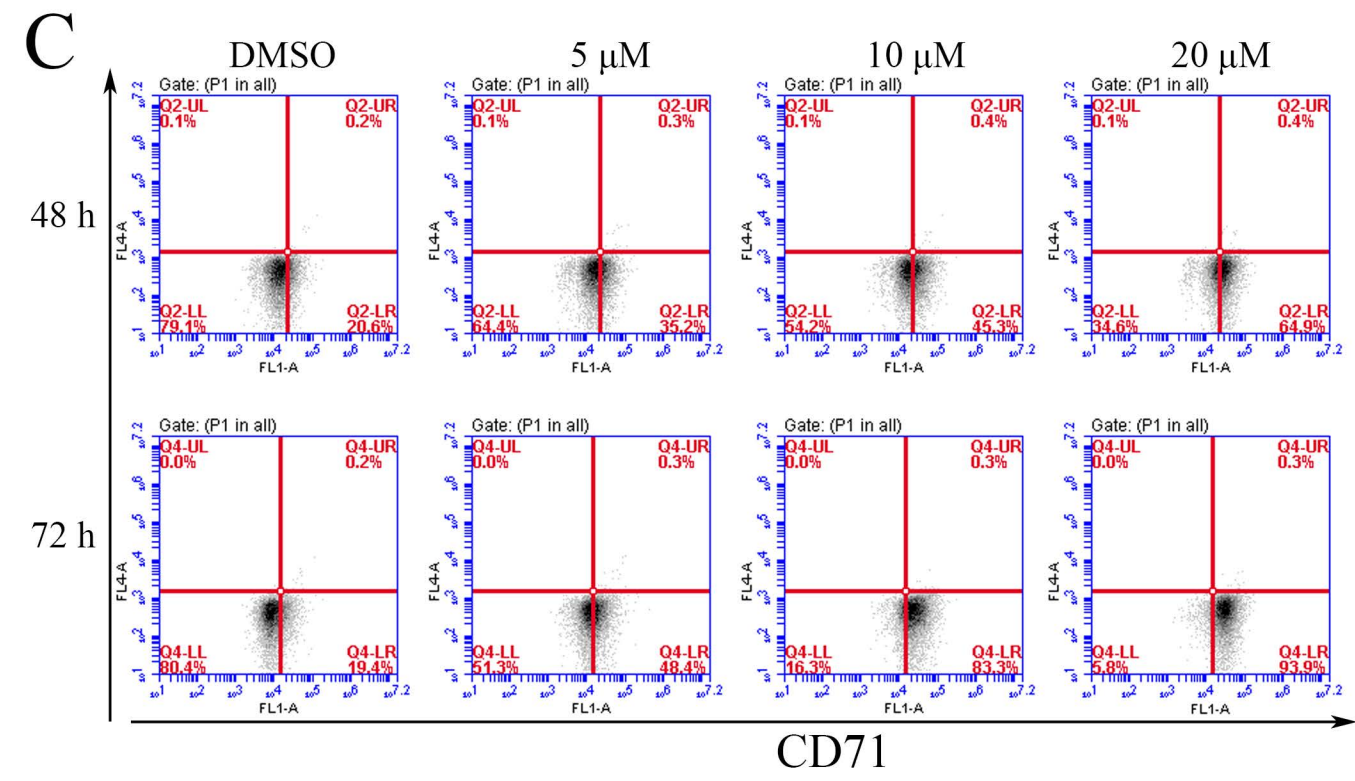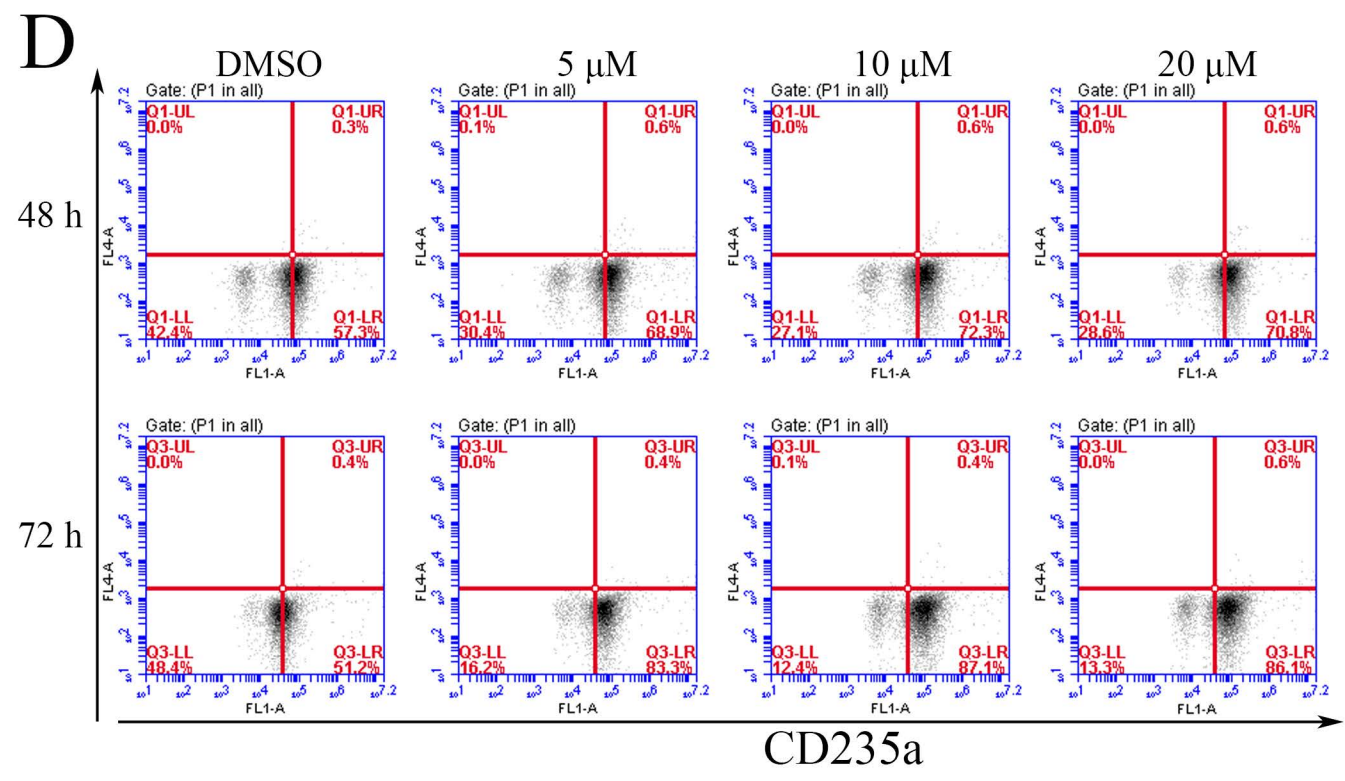

Supplement: Supplementary file 1 [file pharmaceuticals-17-00628-s001.zip › Supplementary Figure S1.pdf]
